# Prenatal Ultrasound Screening: False Positive Soft Markers May Alter Maternal Representations and Mother-Infant Interaction

**DOI:** 10.1371/journal.pone.0030935

**Published:** 2012-01-23

**Authors:** Sylvie Viaux-Savelon, Marc Dommergues, Ouriel Rosenblum, Nicolas Bodeau, Elizabeth Aidane, Odile Philippon, Philippe Mazet, Claude Vibert-Guigue, Danièle Vauthier-Brouzes, Ruth Feldman, David Cohen

**Affiliations:** 1 Department of Child and Adolescent Psychiatry, APHP, GH Pitié-Salpétrière, Paris, France; 2 CNRS UMR 7222, Institut des Systèmes Intelligents et Robotiques, Université Pierre et Marie Curie, Paris, France; 3 Service de Gynécologie Obstétrique, Groupe Hospitalier Pitié-Salpêtrière, APHP, Université Pierre et Marie Curie, Paris, France; 4 Laboratoire de Psychopathologie et de Psychologie Médicale, Université de Bourgogne, Dijon, France; 5 Gonda Brain Research and Psychology Department, Bar Ilan University, Tel Aviv, Israël; Institute of Psychiatry at the Federal University of Rio de Janeiro, Brazil

## Abstract

**Background:**

In up to 5% of pregnancies, ultrasound screening detects a “soft marker” (SM) that places the foetus at risk for a severe abnormality. In most cases, prenatal diagnostic work-up rules out a severe defect. We aimed to study the effects of false positive SM on maternal emotional status, maternal representations of the infant, and mother-infant interaction.

**Methodology and Principal Findings:**

Utilizing an extreme-case prospective case control design, we selected from a group of 244 women undergoing ultrasound, 19 pregnant women whose foetus had a positive SM screening and a reassuring diagnostic work up, and 19 controls without SM matched for age and education. In the third trimester of pregnancy, within one week after delivery, and 2 months postpartum, we assessed anxiety, depression, and maternal representations. Mother-infant interactions were videotaped during feeding within one week after delivery and again at 2 months postpartum and coded blindly using the Coding Interactive Behavior (CIB) scales. Anxiety and depression scores were significantly higher at all assessment points in the SM group. Maternal representations were also different between SM and control groups at all study time. Perturbations to early mother-infant interactions were observed in the SM group. These dyads showed greater dysregulation, lower maternal sensitivity, higher maternal intrusive behaviour and higher infant avoidance. Multivariate analysis showed that maternal representation and depression at third trimester predicted mother-infant interaction.

**Conclusion:**

False positive ultrasound screenings for SM are not benign and negatively affect the developing maternal-infant attachment. Medical efforts should be directed to minimize as much as possible such false diagnoses, and to limit their psychological adverse consequences.

## Introduction

As screenings and predictive medicine develop, it is important to address the question of its potential secondary effects. Addressing this question is particularly crucial when screenings such as foetal ultrasounds apply to vulnerable persons, for example, pregnant women. In approximately 5% of pregnancies, routine foetal ultrasound screening detects a foetal morphological feature that is not considered to be problematic per se, but requires further diagnostic work-up to establish whether it is a normal variant (false-positive screening) or whether it marks a severe foetal condition such as a chromosomal anomaly (true positive-screening). Such morphological features are referred to as “soft markers”(SM) [1]. SM include increased nuchal translucency or short nasal bone in the first trimester, and hyperechogenic bowel, short nasal bones, renal pyelectasis, intracardiac foci, short femur [2], nuchal fold [3], or mild cerebral ventriculomegaly [4] in the second trimester.

High levels of anxiety and psychological distress have been documented in pregnant women for whom a foetal malformation is suspected [5]–[8], diagnosed [8], [9], or when prenatal diagnosis appears ambiguous [9]. The finding of a SM is also associated with psychological distress and anxiety [10]. However, providing reassurance during the ultrasound scan may reduce such anxiety [11], [12] and this is particularly important as maternal stress and anxiety during pregnancy have been associated with increased risk for depression [13]. Moreover, prenatal stress has been shown to impair the quality of the mother-infant interaction in both animal [14] and human studies [13], [15]. In turn, failure to address maternal stress during pregnancy stress may bear short-term negative consequences for infant development and well-being [7], [13], [16]–[19].

Very little research addressed the effects of prenatal ultrasound revealing a SM followed by a reassuring diagnostic work up on the development of the mother-infant bond. It is not known whether the mother's emotional reactions leads to higher depression or alters the mother's representations of the foetus or newborn. Similarly, no research to our knowledge has examined the effects of SM diagnosed during pregnancy on the developing mother-infant interaction in the early postpartum. The goal of this study was to explore the impact of a false-positive ultrasound diagnosis of a “soft marker” on (1) maternal anxiety and depression during and after pregnancy, (2) maternal representations of the infant during and after pregnancy, and (3) mother-infant interaction during feeding at birth and 2 months postpartum. To achieve this, we conducted a prospective case control study using an extreme-case design (244 women were screened) in which 19 pregnant women whose foetus had a positive SM ultrasound screening were compared to 19 women with negative ultrasound screening, matched for age and education.

## Methods

### Design and Participants

We recruited cases and controls in the Gynaecology-Obstetric Unit of GHU Pitié-Salpêtrière, Paris from November 2004 to April 2005 and from October 2007 to May 2010, during a total of 38 months. Inclusion criterion for cases was prenatal diagnosis of a SM at foetal ultrasound followed by a reassuring diagnostic work-up; the inclusion criterion for controls was an uncomplicated pregnancy. Exclusion criteria, for both cases and controls, were a history of somatic or psychiatric illness, a history of significant morbidity during a previous pregnancy, any severe abnormality diagnosed during the current pregnancy, poor understanding of the study protocol. Single pregnant women, women without health coverage, non-French speaking women, and women younger than 18 or older than 38 were also excluded. The institutional review board (*Comité de Protection des Personnes from the Groupe-Hospitalier Pitié-Salpétrière*) approved the study and both parents gave written informed consent after they received verbal and written information on the study.

**Figure 1:**
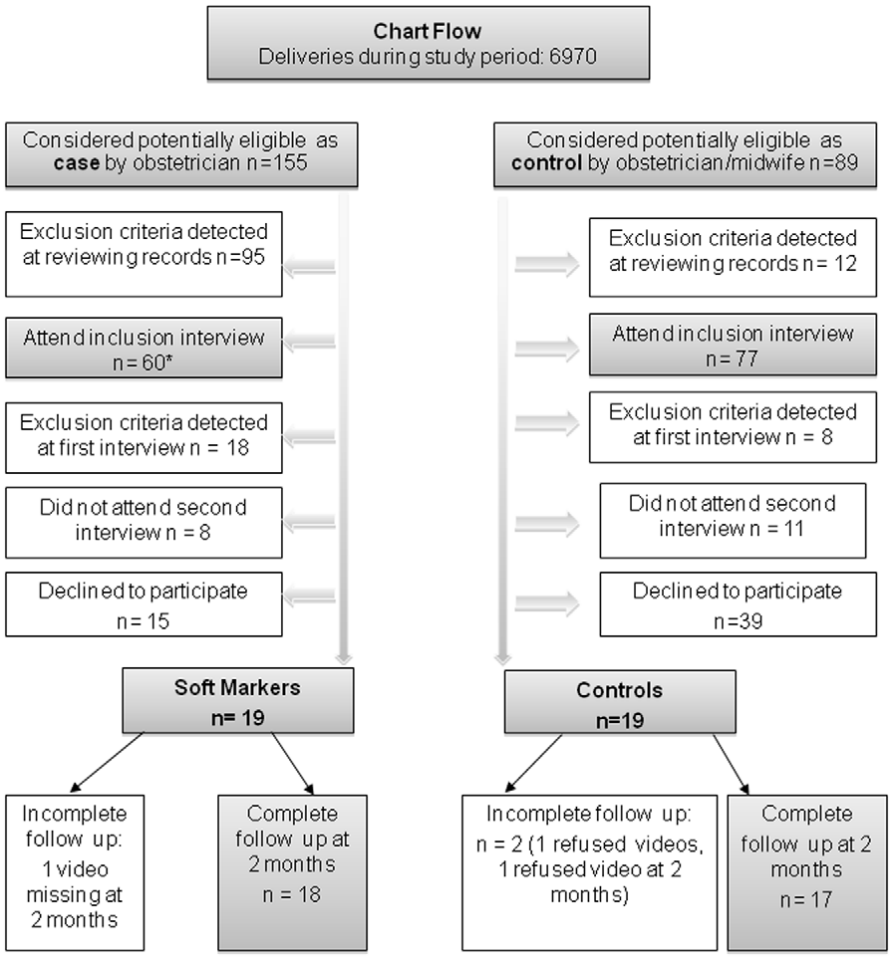
Diagram flow of the study. *Ultrasound soft markers included ventriculomegaly (N = 8), increased nuchal translucency (N = 16), Short OPN (N = 4), echogenic bowel (N = 19), echogenic intracardiac focus (N = 2), mild pyelectasis (N = 9), and short femur length (N = 2).

Of the 6970 tested in the unit during pregnancy, 155 pregnant women were considered as potentially eligible for participation by the perinatologists in charge of prenatal diagnosis, because their ultrasound screenings revealed SM, their following diagnostic work-ups were reassuring, and they were willing to attend a pre-inclusion visit. Of these, 19 met all inclusion criteria eventually, and consented to be enrolled in the study (figure 1). Eighty-nine pregnant women were considered as potentially eligible controls by the obstetrician or midwife in charge of prenatal care based on an uncomplicated pregnancy and willingness to attend a pre-inclusion visit. Of these19 met all inclusion criteria eventually, and accepted to be enrolled (figure 1).

We assessed the comparability of the groups based on demographic and obstetrical variables as well as life events during pregnancy. Life events were analysed using the Sensations During Pregnancy and Life Event Questionnaire [20] (occurrence, severity) as well as general data (parity, socio-demographic status, and medical history) and stressful sensations during pregnancy.

### Outcome Measurements

A clinician (psychologist or child psychiatrist) blind to group status interviewed women in the third trimester of pregnancy, within one week after delivery, and 2 months postpartum. Obstetrical and medical data were recorded and exclusion criteria were assessed at all times (figure 1).

Maternal representations were assessed during pregnancy using the Interview of Maternal Representations during pregnancy (IRMAG) [21] and after delivery using the STERN R Interview [22]. These semi-structured interviews explore maternal representations during pregnancy. The narrative pattern is explored through seven dimensions using a 5-point scale: richness of perceptions, openness to change, affective involvement, coherence, differentiation, social dependence, and richness of fantasies. The final score categorises women's representations in three patterns: good (integrated/equilibrate), intermediate (restricted/disengaged), and poor (non-integrated/ambivalent) either pre or postnatal. Blind analysis was not possible given the impact of SM on maternal representations.

Anxiety was assessed using the COVI scale (maximum score = 12; threshold for disorder = 6) [23]. Depression was assessed using the RASKIN scale (maximum score = 12; threshold for disorder = 6) [24]. DSM-IV-TR symptoms for Major Depressive Episode were systematically assessed when RASKIN score was above the clinical threshold.

Mother-infant interaction was evaluated during breast or bottle-feeding within first week after delivery and at 2 months postpartum. Mothers freely fed their children when they decided to and did so in their natural setting (home). The entire feeding interaction sequence was videotaped. Sessions were analyzed offline using the Coding Interactive Behavior (CIB) Newborn and Feeding Scale [25], [26] which has been validated for coding mother-newborn interactions [27] during play and feeding sessions [25]. Measures of mother-newborn interactions coded with the CIB have shown to predict children's cognitive, social-emotional and neurobehavioral development across childhood [18], [28], [29]. The videotaped feeding interaction was rated by child psychiatrists and developmental psychologists blinded to the perinatal history. Raters received specific training on coding using the CIB. The inter rater agreement measured on ten mother-infant dyads was good (N of raters = 3; kappa = 0.82 [95% Confidence Interval = 0.65–0.98]).

The CIB is a global rating system of parent-child interaction that contains both micro-level codes and global rating scales. Each code is rated from 1 (*a little*) to 5 (*a lot*). Forty-two different codes are grouped into several interactive composites. Five composites were used in the current study as follows: (1) *Maternal sensitivity* was the average of maternal acknowledgment of infant interactive signals, imitation of the infant's behaviour, appropriate tone of voice/motherese, appropriate range of affect, resourcefulness in dealing with infant negative states, supportive presence, dyadic reciprocity and adaptation/regulation of the dyad (Chronbach's alpha = 0.965); (2) *Mother intrusiveness* was the average of maternal inappropriate physical manipulation, mother overriding behaviour (the degree to which mother disregards the infant's signals and interrupts the infant's ongoing behaviour), maternal anxiety, maternal negative affect/anger toward the baby, maternal criticising of infant's behaviour, and mother-led interaction (the degree to which interactions were judged to be led by the mother's needs rather than infant's needs, pace, and agenda) (Chronbach's alpha = 0.867); (3) *Mother-infant positives affect* was the average of the mother's elaboration of the infant's vocalisations and movements, gaze directed to the infant, warm and positive affect, praise of the infant's behaviour, affectionate touch and enthusiasm, and child gaze directed to mother and positive-content affect (Chronbach's alpha = 0.72); (4) *Infant avoidance* was the average of the child's avoidance behaviour toward the mother, the degree to which the infant was uninvolved, non-participating and detached from the feeding activity, and the infant's emotional lability, fatigue, or low level of alertness (Chronbach's alpha = 0.793); (5) *Negative dyadic status* was the average of maternal negative affect/anger, the mother's hostility behaviour, the child's negative emotional affect, dyad constriction, and expression of tension (Chronbach's alpha = 0.793). Composites 4 and 5 were used at two months only.

In addition to the global 42 codes used in prior studies by Feldman and colleagues [25], [26], we used six additional codes for mother and five for infant validated for feeding setting [27]. Maternal codes included holding, confidence in feeding, distractibility, firmness in finished feed, interruptiveness, and quality of post feeding accompaniment. Infant codes included handling, appropriateness of infant state for feeding, easy to suck, distractibility, feeding efficacy. Finally, to assess newborn status based on *Neonatal Behavioral Assessment Scale* of Brazelton [30]: ten items for mother about touch, language and gaze and eight for baby for touch, gaze and vigil state. Feeding and Newborn codes were constructed on the basis of previous research [27], [29]. Five items of Feeding were average into a single composite (Chronbach's alpha = 0.865).

### Statistical analysis

Analyses were carried out using the R package, version 2.10. All tests were two-tailed with p values<0.05 considered significant. We computed descriptive statistics for socio-demographic and clinical characteristics of the SM group and control groups. We used Fisher's exact test to compare qualitative variables (socio-demographics, medical and obstetrical history, maternal representations, delivery and infant characteristics, and feeding practices). For continuous variables that were normally distributed, we used a Student's t-test for between-group comparisons; in the case of a non-normal distribution, the non-parametric Mann-Whitney test was applied. To limit type I error due to multiple comparisons, we used the Holm correction.

To assess association between depression and anxiety scores at inclusion and CIB composite scores at 2-month postpartum, we used Spearman correlation coefficient. To assess whether group differences in 2-month postpartum CIB scores were independent of anxiety or depression scores at inclusion, we analysed CIB composite scores at 2 months postpartum with an ANCOVA after adjusting successively for anxiety or depression scores at inclusion (anxiety and depression scores could not be entered at the same time in a multivariate analysis because the two variables were correlated). The hypothesis of equal slopes was checked (no interaction between covariate and factor) and Pearson residuals were used to assess the model fit. Finally, a cumulative link mixed model was used to check the relationship between the maternal representation variable (3 levels ordered factor) and the levels of anxiety and depression at each time of measurement.

## Results

**Figure 2:**
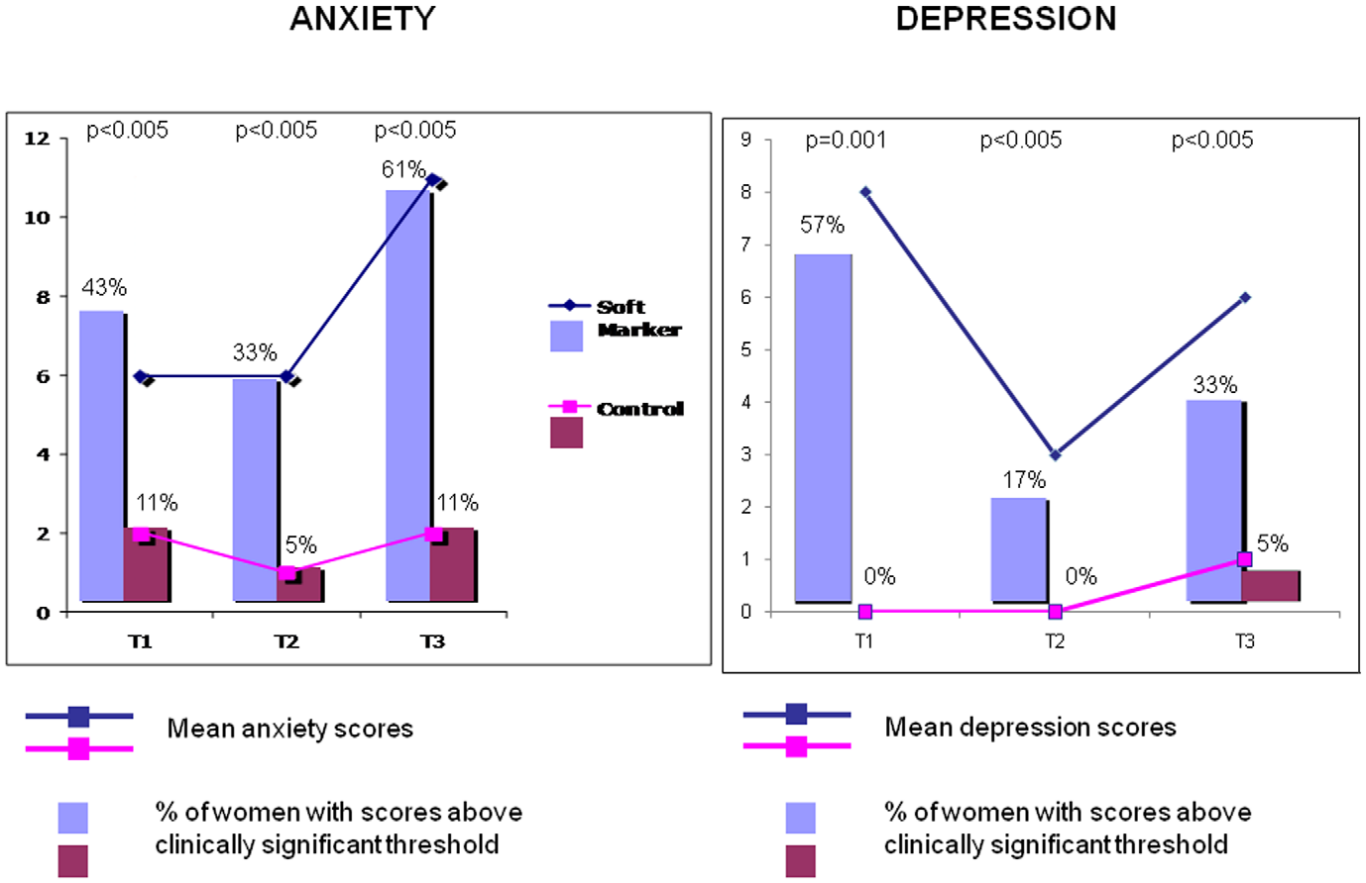
Maternal anxiety and depression over time. Mean scores are given for anxiety and depression (lines). Percentages indicate the number of participants with anxiety (or depression) scores above the scale clinical threshold (bars). T1 = Third trimester during pregnancy, T2 = Birth, T3 = 2 months after birth.

As seen in Table 1, the SM and control group did not differ on socio demographic and obstetrical conditions or on stress and life events during pregnancy. Life events numbers scored by the stress events questionnaire were comparable in the two groups. However, mean anxiety and depression scores were significantly higher in the SM group as compared to controls. The percentages of women with anxiety scores above the COVI scale threshold or with depression scores above the RASKIN scale threshold were significantly greater in the SM group than in the control group. The difference between cases and controls tended to increase at 2 months post partum (Figure 2). Maternal representations in the SM group were more frequently affected, with more intermediate (reduced/loss involvement) and poor (non-integrated/ambivalent) representation patterns observed at all times.

**Table 1:** Socio-demographic, pregnancy, delivery, newborn, and dyad characteristics according to scan soft markers or not.

	Soft Markers (N = 19)	Control (N = 19)	*p*
*Socio-demographic Characteristics*			
Mother's age (years): mean (±SD)	32.3 (±4.2)	32.2 (±3.9)	0.912
Couple Status: unmarried / married	61% / 33%	57% / 42%	0.737
Education Level: Completed A-level vs. Some University vs. Completed University	<5% vs. 11% vs. 83%	<5% vs. 31% vs. 63%	0.328
*Pregnancy Characteristics*			
Minor Obstetrical History*	50% yes / 50% no	36% yes/64% no	0.635
Minor Medico-chirurgical History*	27% yes / 73% no	31% yes / 69% no	1
Para	0.7 (±0.8)	0.9 (±0.7)	0.624
Gesture	1.9 (±0.9)	2.1 (±1.1)	0.733
Life Events Number	7.57 (±3.5)	8.43 (±4.8)	0.595
*Delivery Characteristics*			
Type of delivery: % Vaginal	66%	95%	0.06
Type of delivery: % Caesarean section	33%	5%	0.075
Gestational age	41.47 (±1.5)	41.15 (±2.2)	0.6
*Maternal representation of the baby (good/intermediate/poor)* **			
Third trimester	1/8/9	17/2/0	<10^−5^
Birth	1/9/8	18/1/0	<10^−5^
2 months postpartum	0/12/6	17/1/0	<10^−5^
*Newborn Characteristics*			
Infant Gender: Boy vs. Girl	66% vs. 33%	68% vs. 32%	1
Weight (g)	3483.95 (±376.3)	3348.33 (±551.5)	0.386
APGAR score 5′	10	10	1
*Feeding Practices*			
Bottle	44%	10%	0.053
Breast feeding one week and stop	11%	26%	
Breast feeding until 2 months	44%	63%	

*Given our exclusion criteria, participants had only minor obstetrical histories (e.g., IVG, caesarean for previous pregnancy) or minor medico-chirurgical histories (e.g., appendicitis, minor allergy).

**Good = integrated/equilibrate; intermediate = reduced/loss involvement; poor = non-integrated/ambivalent.

**Figure 3:**
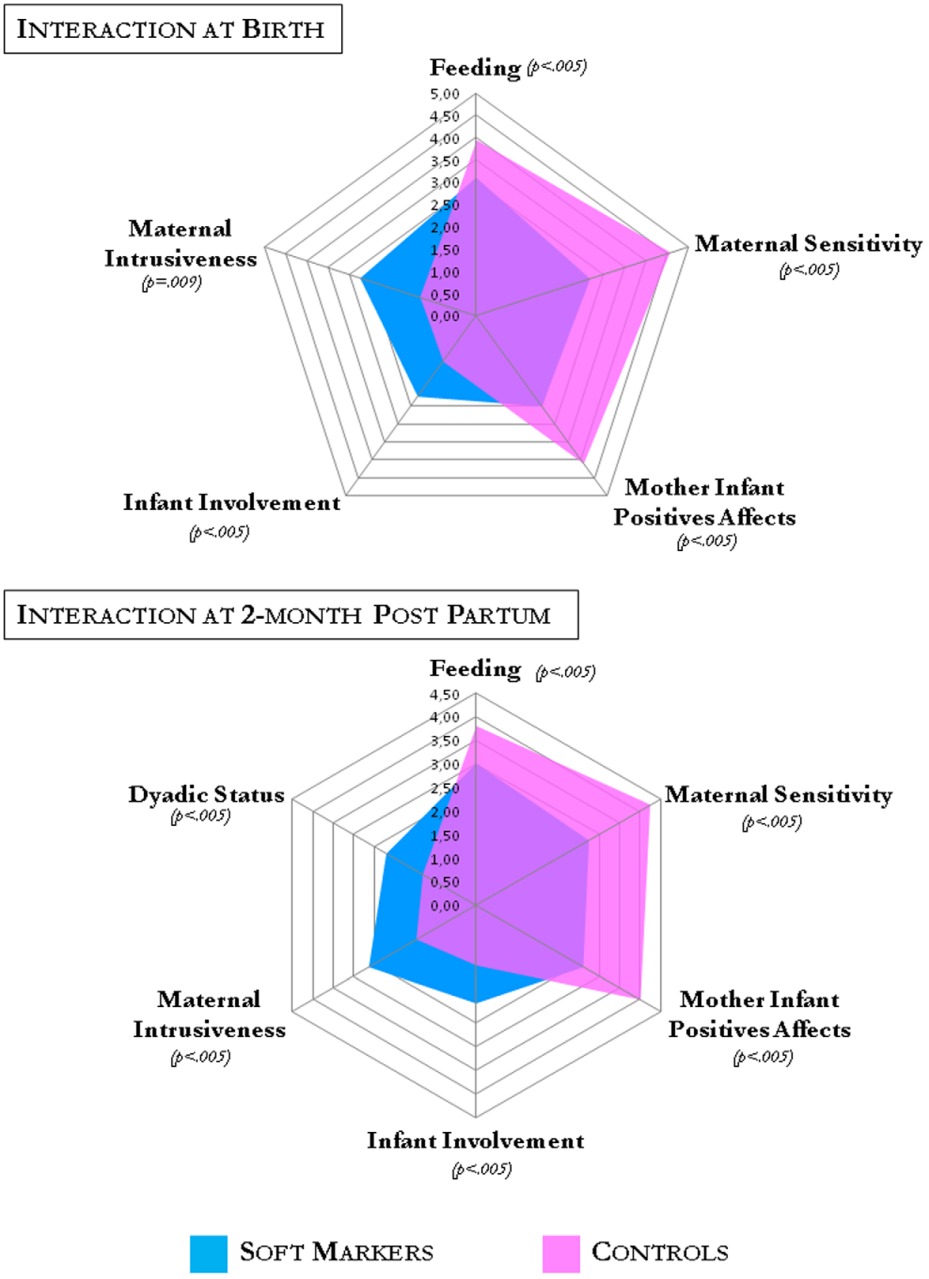
Mother-infant interaction at birth and 2 months postpartum. Mean composite scores are given from the Coding Interaction Procedure.

Feeding practices differed between groups, with a higher rate of bottle feeding among SM cases at childbirth (44% vs. 10%) and 2 months postpartum (56% vs. 37%). CIB analysis after birth and 2 months post partum showed differences in mother-infant feeding quality. Maternal sensitivity and mother-infant positive affects were significantly lower, whereas mother intrusiveness, negative dyadic states, and infant avoidance were significantly higher in the SM group compared to controls. Furthermore, the gap between SM cases and controls tended to increase during the first 2 months postpartum for the following items: maternal sensitivity, mother intrusiveness, mother-infant positive affect, infant involvement, dyadic negative states, and feeding. Figure 3 summarises the CIB composite scores at birth and at 2 months postpartum.

Overall, these findings indicate that following a false positive SM mothers were less sensitive, had difficulties perceiving and elaborating their infants' signals, and their vocalizations to the baby were often inappropriate. They showed limited flexibility in engaging with their infants. In turn, the infant's signals and behaviour were less elaborate and they were less involved in the interaction. Mothers expressed fewer positive emotions towards the infant and interactions were more frequently characterised by a depressed mood. Mothers were more intrusive by touch and behavioural patterns and led the interaction according to their agenda rather than attending to the infant's pace and rhythms.

Infants from the SM group showed fewer positive emotions and initiative behaviour toward mother. Infant avoidance of mother was observed more frequently at 2 months and some active withdrawal could be seen in several dyads. Infant were more tired and appeared more fatigued. On the other hand, in both groups infants showed adequate development at birth. Even when they showed avoidance of mother, infants had a good contact with the environment and the observer (eye-to-eye contact, look to the movie camera). Within the dyad, negative dyadic status was more frequent in the SM group; in this group, dyad expression was restricted and anxious, with less emotion and behaviour regulation, less dyadic reciprocity and less dyad adaptation/regulation.

We found a significant correlation between anxiety scores at inclusion and 2 months postpartum CIB composite scores for maternal intrusiveness (ρ = 0.4, p = .03) and feeding (ρ = −0.4, p = .03). Significant correlation also emerged between depression scores during pregnancy and 2 months postpartum CIB composite scores for maternal intrusiveness (ρ = 0.4, p = .029), negative dyadic status (ρ = 0.41, p = .026), and feeding quality (ρ = −0.44, p = .014). ANCOVA analyses were performed to assess whether the between-group differences in CIB scores at 2 months postpartum were mediated by anxiety or depression scores during pregnancy. After adjusting for anxiety or depression, significant differences remained for the CIB composite scores between the two groups.

Anxiety had a significant effect on maternal representations independent from the effect of time: a 1-point increase on the COVI scale doubled the risk of changing maternal representation categories to the negative (estimate = 0.68, p = 0.0025). Depression had no effect on maternal representation category (estimate = 0.26, p = 0.19). ANCOVA analyses were performed to assess whether CIB composite scores at birth and 2 months postpartum were predicted by maternal representation category in the third trimester after adjusting for anxiety then depression. At birth, all CIB scores were significantly correlated with intermediate (reduced/loss involvement) and poor (non-integrated/ambivalent) maternal representations (10^−5^<p<0.03); the feeding composite score was significantly associated with anxiety (p = 0.037); and maternal intrusiveness, maternal sensitivity, and feeding composite scores were significantly associated with depression (all p<0.016). At 2 months postpartum: all CIB scores were significantly associated with intermediate (reduced/loss involvement) and poor (non-integrated/ambivalent) maternal representations (0.0002<p<0.05); the feeding composite score was only significantly associated with depression (p = 0.036).

## Discussion

The current study is the first to show that SM detected in foetal scan during pregnancy and false positive ultrasound screening increases maternal anxiety and depression symptoms up to 2 months postpartum. It also has a negative impact on the mother's representation and early mother-infant interaction. These finding may have significant clinical and ethical implications. In many developed countries, ultrasound screening is routinely offered to all pregnant women; yet, in up to 5% of pregnancies, a minor foetal anomaly or SM is identified. For example, fetal nuchal translucency measurement greater than the 98^th^ percentile, hyperechogenic fetal bowel, mild pyelectasis, moderate cerebral ventriculomegaly, intracardiac foci are reported to be detected in respectively 2%, 0.5%, 1%, 0.78%, and 2% of pregnancies [2]–[4]. Even if prenatal diagnosis is eventually reassuring, our results show that this is not a benign procedure and may place the mother at higher risk of experiencing a negative emotional reaction and altered representations of her baby. As a result, the infant may be at greater risk for experiencing less optimal maternal care, lower maternal sensitivity and this may carry short-term [31] adverse developmental consequences [13], [32], [33].

These results should be discussed in light of potential biases. First, we only compared 19 dyads per group due to high rates of study refusals. Second, per se, the fact that only a portion of potentially eligible women eventually consented to enter the study is a also potential bias. Notably, 60% of mothers who were approached declined to participate citing their partner's refusal, particularly of videotaping feeding. However, the declining mothers and infants did not differ from the participating families on demographic and medical conditions, including infant birth-weight and gestational age and parental age and level of education. In addition, our exclusion criteria were stringent and eliminating potential confounding of the findings: the experiment and control groups were carefully case-matched on maternal education, marital status, pregnancy history, and concurrent stress and life events. The fact that mothers in the SM group were less inclined to breastfeed their babies may be one adverse outcome of their prenatal ultrasound experience. Given that breastfeeding has shown in numerous studies to be beneficial to children's cognitive, emotional, and neurobehavioral development and to promote a more positive mother-child relationship [34]–[36], this may be one negative outcome of the false positive SM detection. Furthermore, among controls, rate of postpartum depressive symptoms was similar to what is expected in the general population after pregnancy [31], [37], [38]. Other potential biases include: (1) videotaping that was not blind to group status leading to modifications of spontaneous maternal behaviours. (2) Although clinical and video assessments were blind, it was not possible to maintain blind assessment of maternal representations. However, this was a direct consequence of the impact an SM had on maternal representation, and most mothers in the SM group referred to the SM diagnosis during the semi-structured interviews. Finally, the heterogeneity of conditions associated with soft markers limits the generalization of our results. Paediatric outcome after prenatal diagnosis of pyelectasis is nearly universally good. In contrast, cerebral ventriculomegaly or increased nuchal translucency may be associated with severe neurological or genetic conditions even though prenatal diagnostic work up is reassuring. In future studies, maternal outcomes should be specifically related to each prenatal ultrasound finding.

Indeed, previous studies have shown that maternal stress, depression, and anxiety increase during pregnancy after a foetal SM has been detected [10], [11], but so far the impact of a false positive ultrasound screening on the mother's pattern of representation and early interactions has not been previously evaluated. One intriguing result of this study is that anxiety and depression scores remained high, and less optimal mother-infant interaction patterns persisted at 2 months postpartum despite the infants' normal development. In contrast, maternal representations remained altered at all time-points and appeared to be the strongest predictor of the evolving mother-infant interactions. We conclude that anxiety and depression are broad indicators of the maternal state with a good sensitivity and cannot be considered specific of the ultrasound prenatal screening. The fact that the percentages of participants with anxiety and depression scores above the COVI and RASKIN scale clinical thresholds were lower at birth than at 2 months postpartum, in both groups, may be explained by natural development that occurs in the period surrounding birth and delivery [39]. We hypothesise that maternal representations may be a more proximal mediator of the effect that SM detection has on the mother's emotional reaction and mother-infant interaction.

The persistence of a less optimal mother-infant interaction at 2 months postpartum is consistent with the attachment, interactional, and psychodynamic theories that hypothesise that early interactions are constructed through the mother's mental representations of her baby, as well as the mother's confidence in her maternal abilities and in the infant's abilities to develop [16], [17]. Guided by these perspectives, we may hypothesise that the prenatal diagnosis of an “abnormal” foetal mark may disrupt the formation of the maternal bonding-related representations. Mothers may experience suspension of their invested in the infant and in the development of a more vivid and detailed representations of the attachment relationship. The way they imagine the future of their infant would be altered, resulting in a tense infant-mother meeting at birth [29], [40]. It is difficult to determine whether the difference in mother-infant interaction between the SM and control groups would have an impact on child development. However, previous studies have shown the impact of early mother interaction/synchrony patterns on infant development such as symbolic play and internal state talk at 2 years [41], attachment security at 1 year [18], and later adolescent's capacity for empathy and moral orientation [42].

What may be the practical implications of the current findings? By no means should prenatal screening be abandoned. A large corpus of evidence shows that prenatal screening for foetal anomalies meets the expectations of pregnant women [43] and that a negative result at screening has a reassuring impact on pregnant women [44]. Several studies demonstrated that ambiguity concerning diagnosis or prognosis induces a particular acute distress [9], [10]. Sonographers, midwives, and perinatologists should try to present clear, reassuring information to the parents. We also believe that healthcare professionals at large should be informed that false positive foetal ultrasound screening might alter early mother-infant interaction. This could help obstetricians, midwives, and sonographers in diagnosing and managing anxiety, depression, or altered mother-infant interaction in women whose pregnancy was marked by the finding of an SM. Paediatricians and general practitioners should consider false positive ultrasound screening to be a significant prenatal event. Given that anxiety and depression auto questionnaires have been validated for use during pregnancy [15], [43], we recommend systematically screening for anxiety and depression at the visit immediately following SM detection to identify at-risk women and offer psychological treatment. Indeed, the ideal time to begin mental health care could be during the obstetric follow-up, when the psychic dynamics of the woman are accessible, or immediately in postpartum, while the mother is still in the hospital and the dyadic pattern is establishing [18], [19]. Psychologists and psychiatrists should bear in mind the potential impact of such prenatal events when dealing with psychological problems in mothers or their offspring, and during their therapeutic approach should focus on maternal representation, as it appear to be a key mediating factor.

In conclusion, our results suggest that there may be a gap between the way the foetal ultrasound scan is generally represented as “harmless” and its potential impact on both the psychological state of the pregnant mother and mother-infant interaction. We found that the impact of a false positive ultrasound screening persists after birth until 2 months postpartum. Given the frequency with which foetal scan are used to detect at-risk pregnancies, preventative measures should be recommended in case of SM detection, in particular when pregnant women express high emotional distress after SM diagnosis.
