# Correction: Prenatal Ultrasound Screening: False Positive Soft Markers May Alter Maternal Representations and Mother-Infant Interaction

**DOI:** 10.1371/journal.pone.0091494

**Published:** 2014-03-10

**Authors:** The PLOS ONE Staff

An error occurred in the Figure 1 legend. The correct legend should read: "*Ultrasound soft markers included mild ventriculomegaly (N  =  3), increased nuchal translucency (N  =  3), short nasal bones (N  =  1), hyperechogenic bowel (N  =  7), pyelectasis (N  =  5)."

**Figure 1:**
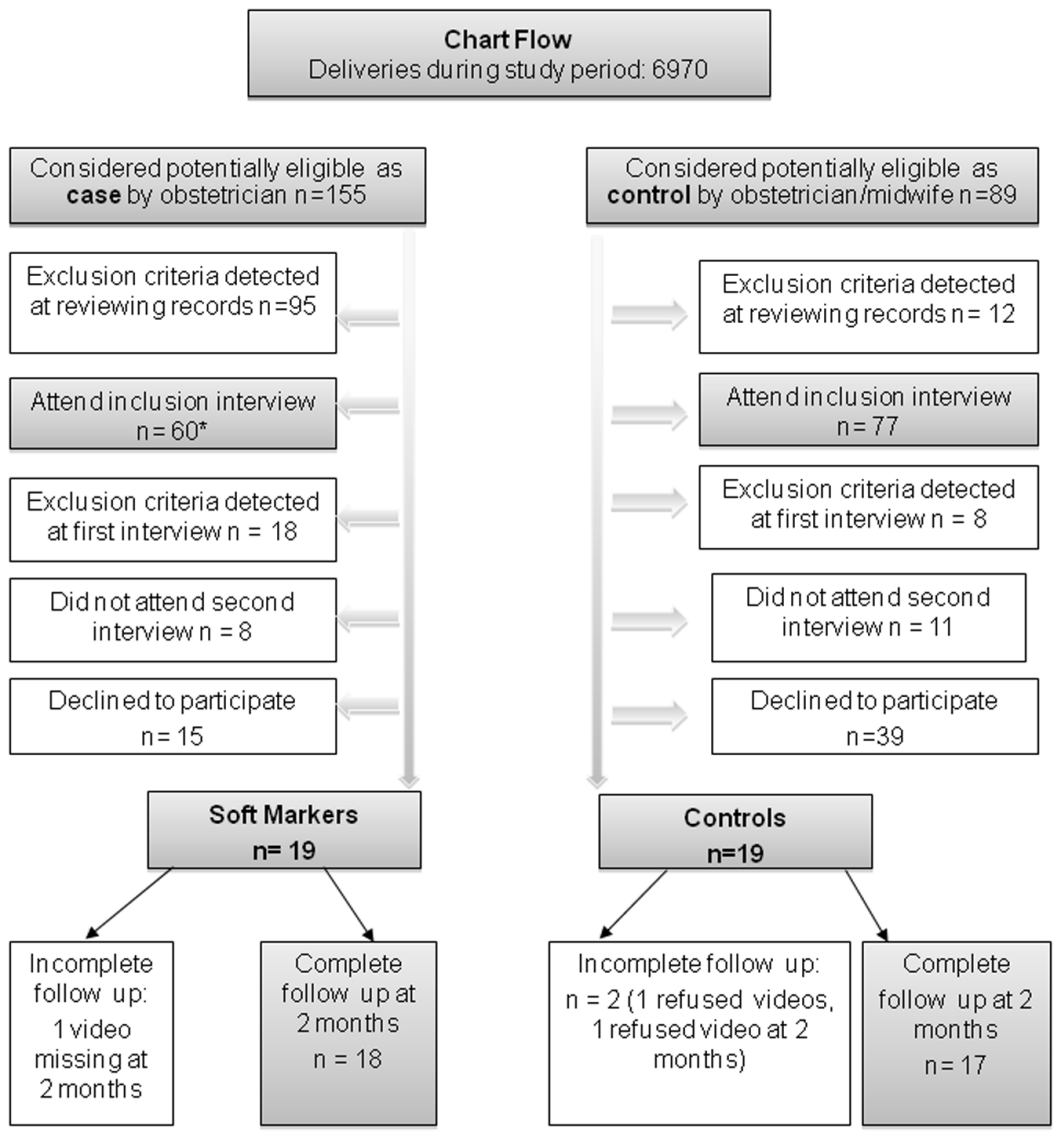
Diagram flow of the study. Ultrasound soft markers included mild ventriculomegaly (N = 3), increased nuchal translucency (N = 3), short nasal bones (N = 1), hyperechogenic bowel (N = 7), pyelectasis (N = 5).
